# Benefits of Mobile Contact Tracing on COVID-19: Tracing Capacity Perspectives

**DOI:** 10.3389/fpubh.2021.586615

**Published:** 2021-03-18

**Authors:** Uichin Lee, Auk Kim

**Affiliations:** ^1^School of Computing, Korea Advanced Institute of Science and Technology, Daejeon, South Korea; ^2^Department of Computer Science and Engineering, Kangwon National University, Chuncheon, South Korea

**Keywords:** COVID-19, contact tracing, mobile contact tracing, contact tracing app, contact tracing capacity modeling, digital contact tracing

## 1. Introduction

For effectively suppressing COVID-19's spread, contact tracing has been widely used to identify, isolate, and follow-up with those who have come in close contact with an infected person (or “close contacts”). Traditionally, contact tracers in local health offices interview an infected person to identify visited places (or hotspots) and then check any close contacts. For the accurate recall of travel history, several countries including South Korea corroborate multiple data sources, such as cell location or credit card transactions (1).

Beside this traditional approach, various mobile apps were introduced to help improve travel history tracking including automated GPS tracking (e.g., Israel's HaMagen) and manual place QR-code scanning (e.g., New Zealand's NZ COVID Tracer and Korea's KI Pass). Alternatively, mobile apps maintain individuals' “encounter history” (instead of place visit history) by leveraging peer-to-peer wireless beaconing (i.e., self-announcing its presence to nearby devices) with Bluetooth Low Energy (BLE) in smartphones, such as Google-Apple's Exposure Notification and Singapore's BlueTrace and TraceTogether. This encounter history can be used later to judge whether a user had a risky encounter with an infected person.

We argue that traditional manual contact tracing can be greatly improved by leveraging the wisdom of crowds. Local community members install mobile apps to self-collect “breadcrumbs” for contact tracing, such as GPS traces, place QR-codes, and wireless encounter histories, which can offer near real-time assessment (2). However, there is a systematic lack of adoption of mobile apps in many countries, and success stories of mobile apps are scarce. This is partly because local health authorities are unsure about the benefits of mobile apps, mostly worrying that a lack of app adoption fails to achieve digital herd immunity (3). We emphasize the importance of adopting contact tracing apps via “contact tracing capacity modeling.” Our model clearly demonstrates that the adoption of contact tracing model apps can potentially increase contact tracing capacity. Furthermore, this capacity increment thanks to crowd participation will greatly help local authorities to better handle confirmed cases in the early or late phases of the pandemics. In the following, we present our approach of contact tracing capacity modeling and its benefits for contact tracing. We then discuss practical challenges related to application adoptions by citizens and health authorities as well as technical implementation issues.

## 2. Contact Tracing Capacity Modeling

**Figure 1:**
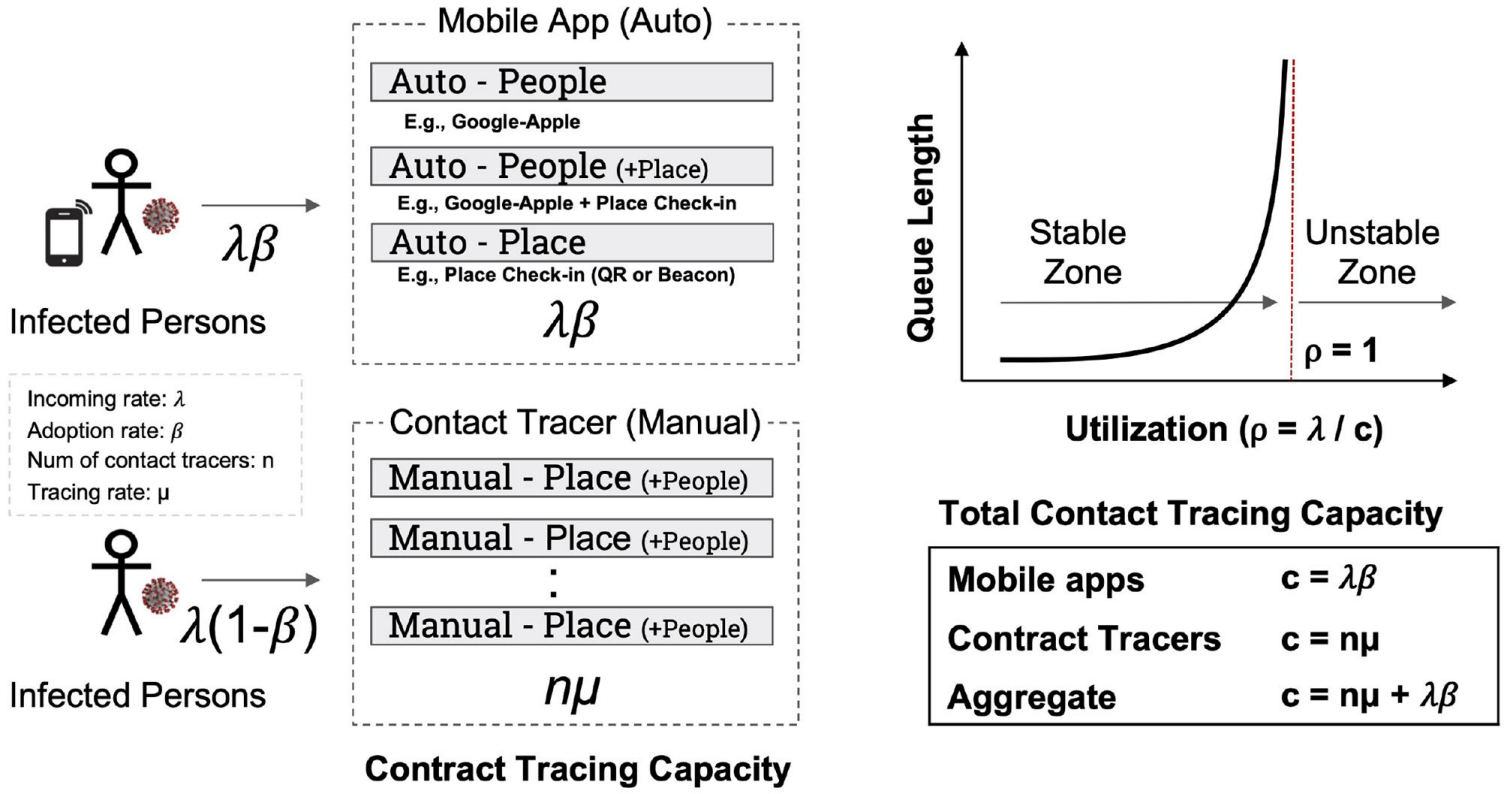
Contract-tracing capacity modeling.

A limited resource model is considered (Figure 1): for the local health authority, there are *n* contact tracers, each having a fixed processing rate μ (e.g., on average, this person can deal with μ people per day). Assuming independence, the total contact tracing capacity would be simply given as *nμ*. The contact tracers may use existing infrastructure including cell location data or GPS apps to improve their processing rate μ (and lower the error rates).

Let's assume that newly diagnosed people are visiting the authority at the rate of λ, which may vary over time. When both incoming and processing patterns follow exponential distributions (as in M/M/n queue) (4), the number of waitlisted people is proportional to system utilization ($\rho =\frac{\lambda }{n\mu }$), and it is represented as $\frac{\rho }{1-\rho }$. If utilization is close to 1, the number of people waiting would dramatically increase, and tracing would be significantly delayed. If local authorities keep the same number of contact tracers or do not sacrifice the quality of contact tracing, there is only a limited number of incoming people for whom contact tracing can be performed; the rest should be excluded due to tracing delay. Therefore, we can easily find that any existing contact tracing system is susceptible to overloading. If a mass infection happens due to super spreading events (e.g., church gathering or indoor parties), overloading could lead to a suspension in normal contact tracing. From a utilization perspective, this phase transition is quite evident in mass infection scenarios.

Fortunately, the adoption of contact tracing mobile apps could help increase the overall contact tracing capacity as follows. When a mobile app's adoption rate is β, the incoming rate of infected persons with an app would be λβ. If the app supports automatic identification of close contacts (which are narrowcast to the target users via the app), the overall contact tracing capacity (without considering its effectiveness) increases from *nμ* to *nμ*+λβ. This kind of capacity scaling due to crowd participation (or crowdsourcing) is also observed in peer-to-peer file sharing, such as BitTorrent, where the service capacity for file downloading linearly increases with the number of participants (5).

## 3. Why Is It Beneficial?

In normal operations where the incoming infection rate is manageable (i.e., stable zone), mobile apps' value is less obvious, particularly when the contact tracers can have infrastructure support including cell location data and credit card transactions for accurate contact tracing, as in Korea. During a massive outbreak, however, the contact tracers' utilization quickly increases to the maximum, and the system becomes unstable. In both situations, mobile apps' adoption is beneficial because early quarantine notice via the mobile app's narrowcasting to exposed people helps prevent further virus spreading. In the unstable zone, the incoming rate of new infection rapidly increases. If mobile apps are adopted, the overall tracing capacity also increases proportionally (i.e., λβ where β is the adoption rate). In the early or late stages of virus spreading, capacity increment helps local authorities to better handle each confirmed case (i.e., fast identification of close contacts before it is too late). Our concept of capacity scaling provides an alternative view on epidemic dissemination modeling where mobile app adoption rate of β helps reduce the infection rate (*R*_0_) by a factor of 1−β^2^ because two parties should have their apps installed for suppression (6).

## 4. Practical Challenges

There are a few practical challenges in implementing and adopting such mobile apps. A good news is that recent studies show that a majority of people are generally willing to install mobile apps [e.g., 74.8%, as per Altmann et al. (7)]. Of course, there are differences across different counties, and some countries, such as Australia show lower rates of adoption (e.g., 37% downloaded, 19% intended, 28% refused, and 16% undecided). High adoption rates are related to the fact that during pandemics, people are willing to trade their privacy for personal and public safety (e.g., disclosing location traces) if they saw clear benefits (8, 9). This kind of altruistic behavior was also observed in a recent study on wearable data tracking campaigns in Germany (10).

However, adoption rates considerably drop if detection accuracy is low (8, 11). For accuracy measurements of different tracing technologies, we can consider precision (how precise is it to identify visits or encounters?) and recall (how accurately does it capture all visits or encounters?). We note that one size does not fit all, and different technologies have different precision/recall characteristics. Cell ID tracking can only offer kilometer level location accuracy (low precision and recall). Fine-grained location tracking is feasible with GPS, but it does not work indoors, such as a large department store (low indoor recall). Place check-in or wireless beaconing can precisely identify place visits or people encounters, but voluntary participation (e.g., manual QR-code scanning, mobile app adoption) is required. Furthermore, according to a recent study in Australia (11), major barriers of COVIDSafe app adoption included privacy concerns (and distrust toward surveillance), technical difficulties, and misbeliefs about the app's operations (data management and key features). The authors noted that privacy concerns could be amplified due to misbeliefs on data management or features; e.g., data could be shared after the pandemic ends or the app can inform whether it is safe to leave the house (11).

Additionally, there are practical implementation challenges of contact tracing technologies. The first is technical implementation issues. Currently, only Google-Apple API supports full operations of “background” contact tracing (12). Various battery optimization techniques are employed in recent smartphones, which tend to remove background applications (e.g., clearing unused apps from active states). Lack of proper control on battery optimization setting may lead to failure in collecting contact trace information using smartphones. Another implementation challenge is rather infrastructural due to a lack of digital transformation in legacy contact tracing systems (13). This may be because the digital acquisition of administrative data is lagging behind due to a lack of human resource training or integration into existing work tasks.

The current modeling argues that mobile apps can potentially increase contact tracing capacity. This argument assumes that mobile technologies can be seamlessly integrated into existing contact tracing practices in local health authorities. This assumption is challenged not only by a lack of digital transformation in the health authorities' work practices, but also by a diversity of needs in pandemic handling. For example, often times, health authorities want a sense of control on mobile contact tracing (e.g., centralized data collection and information sharing), but this need conflicts with the privacy-by-design principle in Bluetooth-based contact tracing mobile apps (i.e., decentralized data collection and anonymized data sharing). Privacy protection is an important aspect to assure, but people are often willing to sacrifice their privacy as long as it helps to contact tracing during pandemics (9).

## Author Contributions

UL wrote the manuscript. AK provided the detailed feedback for paper organization and visualizations. All authors contributed to the manuscript submission, read, and approved the submitted version.

## Conflict of Interest

The authors declare that the research was conducted in the absence of any commercial or financial relationships that could be construed as a potential conflict of interest.
